# Supplementary material for: WordCommentsAnalyzer: A windows software tool for qualitative research
Source: F1000Res. 2018 Sep 4;7:536. Originally published 2018 May 3. [Version 2] doi: 10.12688/f1000research.14819.2 (PMC6137411; doi:10.12688/f1000research.14819.2)
Supplement: Supplementary file 3 [file f1000research-7-17614-s0002.tgz › b2a79496-4ada-47c4-9682-a99ce867836d.zip!/Eight Iranian Health Journals/Archives of Rehabilitation_27.docx]

© Archives of Rehabilitation. This work is licensed under CC BY-NC 4.0 (https://creativecommons.org/licenses/by-nc/4.0/).

Volume & Issue: دوره 13 - شماره 2

Title: بررسی تکرارپذیری اندازه‌گیری قوس، دامنه خم‌شدن به جلو و خم‌شدن به عقب ناحیه کمر با استفاده از دستگاه Dual Inclinometer در افراد سالم و افراد مبتلا به کمردرد مزمن غیراختصاصی

http://rehabilitationj.uswr.ac.ir/article-1-1001-fa.html

هدف: اندازه‌گیری درست قوس ناحیه کمر و دامنه‌های حرکتی هم در ارزیابی بیماران و هم در سنجش اثر درمانهای مختلف توانبخشی از ارزش بالائی برخوردار است. هرچند روش‌های مختلفی جهت اندازه‌گیری قوس کمر وجود دارد اما برخی از آن‌ها پرهزینه بوده و برخی دیگر اعتبار چندانی ندارند. هدف مطالعه فوق بررسی تکرارپذیری دستگاه Dual Inclinometer در اندازه‌گیری قوس ناحیه کمر و دامنه حرکتی خم شدن به جلو و عقب توسط آزمون‌گر واحد می‌باشد. روش بررسی: در یک مطالعه متدولوژیک، قوس و دامنه حرکتی ناحیه کمر ۳۰ زن (۱۲ زن مبتلا به کمردرد و ۱۸ زن سالم) توسط دستگاه Dual Inclinometer در سه نوبت اندازه‌گیری شد. دو اندازه‌گیری در یک روز به فاصله یک ساعت برای بررسی تکرارپذیری درون-روز (Within-Day) و نوبت آخر با فاصله یک روز برای ارزیابی تکرارپذیری بین-روزها (Between-Days) صورت گرفت. یافته‌ها: اندازه‌گیری‌های قوس کمر، حرکت خم شدن به جلو و عقب درون روز برای افراد سالم و کمردردی تکرارپذیری بالائی را نشان می‌دهد (مقدار ضریب همبستگی درون روز برای قوس کمر، خم شدن به جلو و عقب به ترتیب %۹۸، %۷۷ و %۶۹ برای افراد سالم و %۹۴، %۹۵ و %۶۹ برای افراد مبتلا به کمردرد بوده است). اندازه‌گیری‌های بین روز نیز برای هر دو گروه تکرارپذیری بالایی را نشان می‌دهد (مقدار ضریب همبستگی بین روز برای قوس کمر، خم شدن به جلو و عقب به ترتیب %۹۶، %۷۰ و %۶۷ برای افراد سالم و %۹۱، %۷۱ و %۶۶ برای افراد مبتلا به کمردرد بوده است). نتیجه‌گیری: نتایج مطالعه حاضر نشان می‌دهد که دستگاه Dual Inclinometer از تکرارپذیری بالایی در اندازه‌گیری قوس و دامنه حرکتی ناحیه کمر برخوردار بوده و می‌تواند به عنوان یک ابزار قابل اعتماد در ارزیابی بیماران و همچنین در بررسی اثر مداخلات مختلف درمانی به کار گرفته شود. مطالعات آتی جهت حمایت نتایج این مطالعه با انتخاب نمونه‌های بیشتر توصیه می‌گردد.

Title: تکرارپذیری روش الکترومیوگرافی عضلات شکم در طی انجام مانورهای شکمی با و بدون انقباض عضلات کف لگن

http://rehabilitationj.uswr.ac.ir/article-1-1032-fa.html

هدف: این مطالعه با هدف بررسی تکرارپذیری روش الکترومیوگرافی برای تعیین فعالیت عضلات شکم در طی انجام دو مانور گود گردن و سفت کردن شکم با و بدون انقباض عضلات کف لگن در افراد سالم و مبتلا به کمردرد مزمن انجام شد. روش بررسی: در یک مطالعه متدولوژیک ثبت سطحی فعالیت الکترومیوگرافی از عضلات شکمی (عرضی شکمی و مایل داخلی و خارجی) در ۲۱ نفر (۱۲ فرد سالم و ۹ فرد مبتلا به کمردرد) که به طور ساده و در دسترس انتخاب شده بودند، صورت گرفت. هر مانور در سه تکرار انجام می‌شد و تکرار تست در همان روز با فاصله زمانی حداقل ۳۰ دقیقه صورت می‌گرفت. آزمون کولموگروف-اسمیرنوف و محاسبه ضریب همبستگی درون گروهی جهت تحلیل داده‌ها استفاده شد. یافته‌ها: نتایج آزمون بیانگر تکرارپذیری بسیار خوب (۹۰-۸۰=ICC) طی مانورهای گود کردن و سفت کردن عضلات شکم به تنهایی و گود کردن همراه با انقباض عضلات کف لگن و همچنین انقباض ایزوله عضلات کف لگن و تکرار پذیری خوب (۸۰-۷۰=ICC) برای مانور سفت کردن شکم همراه با انقباض عضلات کف لگن بود. نتیجه‌گیری: الکترومیوگرافی روشی قابل اعتماد و تکرارپذیر برای بررسی فعالیت ارادی و غیرارادی عضلات شکم در شرایط مختلف طی مانورهای ذکر شده می‌باشد.

Title: ترجمه و بومی‌سازی چهار پرسشنامه بیمار محور ارزیابی درد و ناتوانی عملکردی شانه و بررسی تکرارپذیری نسخه‌های فارسی آنها در ایرانیان مبتلا به درد و ناتوانی شانه

http://rehabilitationj.uswr.ac.ir/article-1-1031-fa.html

هدف: هدف از این پژوهش دستیابی به یک وسیله ارزیابی تکرار پذیر جهت بررسی کیفیت زندگی ایرانیان مبتلا به درد و ناتوانی شانه می‌باشد. روش بررسی: طی یک مطالعه غیر تجربی از نوع متدولوژیک چهار پرسشنامه شانه به زبان فارسی ترجمه و بومی‌سازی شد. 126 زن و مرد مبتلا به درد و ناتوانی شانه برای بررسی تکرارپذیری نسخه فارسی تهیه شده از این پرسشنامه‌های شانه مورد بررسی قرار گرفتند. اطلاعات به‌دست آمده از لحاظ تکرارپذیری مطلق و نسبی در دفعات آزمون و همخوانی درونی خرده‌مقیاس‌ها و همخوانی درونی اعتبار افتراقی در سطح آیتم‌ها مورد تجزیه و تحلیل آماری قرار گرفت. یافته‌ها: همخوانی درونی هر یک از پرسشنامه‌ها مطابق ضریب آلفای کرونباخ محاسبه شده برای اکثر خرده‌مقیاس‌ها در حد بالایی قرار داشت (حداقل۰/۷۰). همبستگی هر آیتم با خرده‌مقیاس خود، بعد از اصلاح همپوشانی، برای آیتم‌های همه خرده‌مقیاس‌ها بالاتر از حد مطلوب ۰/۴۰ بود که همخوانی درونی خوبی را در سطح آیتم‌های این خرده‌مقیاس‌ها نشان می‌دهد. همچنین نتایج تکرارپذیری خوب نسخه‌های فارسی پرسشنامه‌ها را نشان داد. نتیجه‌گیری: با تهیه نسخه‌های فارسی چهار پرسشنامه درد و ناتوانی شانه ابزار‌های ارزیابی چند منظوره در اختیار است که می‌توان به کمک آنها وضعیت سلامتی و تأثیر درمان‌های مختلف را روی بیماران مبتلا به درد و ناتوانی شانه در کشور را مورد بررسی قرارداد

Title: مقایسه فعالیت عضلات عرضی و مایل داخلی شکمی هنگام تمرین گود کردن شکم در دو وضعیت خوابیده به پشت و چهاردست و پا در افراد مبتلا به کمردرد مزمن غیراختصاصی با استفاده از اولتراسونوگرافی

http://rehabilitationj.uswr.ac.ir/article-1-1034-fa.html

هدف: هدف از انجام این تحقیق بررسی تأثیر وضعیت‌های مختلف بدن بر فعالیت عضلات شکمی در افراد مبتلا به کمردرد مزمن غیراختصاصی می‌باشد. روش بررسی: تغییر ضخامت عضلات عرضی و مایل داخلی شکم ۲۰ مرد مبتلا به کمردرد مزمن غیراختصاصی (۹±۳۹ سال) و ۲۰ مرد سالم (۸±۳۷ سال) طی مانور گود کردن شکم در دو وضعیت خوابیده به پشت و چهاردست و پا با اولتراسونوگرافی اندازه‌گیری شد. حاصل تقسیم تغییر ضخامت مایل داخلی بر ضخامت عرضی شکم جهت جداسازی فعالیت این دو عضله محاسبه گردید. روش بررسی: تغییر ضخامت عضلات عرضی و مایل داخلی شکم 20 مرد مبتلا به کمردرد مزمن غیر اختصاصی(9±39 سال) و 20 مرد سالم (8±37 سال) طی مانور گود کردن شکم در دو وضعیت خوابیده به پشت و چهاردست و پا با اولتراسونوگرافی اندازه گیری شد. حاصل تقسیم تغییر ضخامت مایل داخلی بر ضخامت عرضی شکم جهت جداسازی فعالیت این دو عضله محاسبه گردید. یافته‌ها: تغییر ضخامت عضله عرضی شکم در هر دو وضعیت خوابیده به پشت (۰/۰۰۱>P) و چهاردست و پا (۰/۰۰۱>P) بین گروه سالم و بیمار، نسبت تغییر ضخامت عضله مایل داخلی به عرضی شکم در هر دو وضعیت خوابیده به پشت (۰/۰۰۲=P) و چهاردست و پا (۰/۰۰۶=P) بین دو گروه ، تغییر ضخامت عضلات عرضی (۰/۰۱۲=P) و مایل داخلی شکم (۰/۰۰۳=P) بین دو وضعیت خوابیده به پشت و چهاردست و پا در گروه بیمار و نسبت تغییر ضخامت عضله مایل داخلی به عرضی شکم بین دو وضعیت خوابیده به پشت و چهاردست و پا در گروه بیمار (۰/۰۰۱>P) می‌باشند دارای تفاوت معنادار می‌باشند. نتیجه‌گیری: بیماران مبتلا به کمردرد مزمن غیراختصاصی توانایی کمتری نسبت به افراد سالم در انقباض مجزای عضله عرضی از مایل داخلی شکم طی تمرین گود کردن شکم دارند و در بیماران، در وضعیت خوابیده به پشت نسبت به وضعیت چهاردست انقباض عضله عرضی شکم بهتر انجام می‌گردد.

Title: مقایسه نسبت فعالیت میوالکتریک عضله پهن مایل داخلی به پهن خارجی طویل حین اسکات با اداکشن ران در ورزشکاران حرفه‌ای مبتلا و غیرمبتلا به سندرم درد پاتلوفمورال

http://rehabilitationj.uswr.ac.ir/article-1-1038-fa.html

هدف: مقایسه نسبت فعالیت الکترومیوگرافی عضله پهن مایل داخلی (VMO) به پهن خارجی طویل (VLL) حین حرکت اسکات با اداکشن ران در ورزشکاران مبتلا و غیرمبتلا به سندرم درد پاتلوفمورال (PFPS) هدف این تحقیق بود. روش بررسی: در این مطالعه غیر تجربی مورد-شاهدی، 1۶ ورزشکار مرد تیم ملی (والیبال، هندبال و تکواندو) سنین 30-18 سال مبتلا به PFPS با انتخاب هدفمند و 1۶ ورزشکار مرد سالم تیم‌های ملی که براساس متغیرهای وزن، قد، سن، رشته ورزشی و غالب بودن اندام تحتانی همتاسازی شده بودند، به‌ صورت داوطلبانه و براساس معیارهای ورود و خروج شرکت کردند. فعالیت الکترومیوگرافی عضلات VMO و VLL توسط الکترودهای سطحی و سیستمTelemetric EMG حین اسکات با اداکشن ران در زوایای ۱۵، ۳۰ و ۴۵ درجه فلکشن زانو ثبت و نسبت فعالیت عضلات VMO به VLL محاسبه گردید. جهت مقایسه نسبت عضلات مذکور در دو گروه، از آزمون آماری آنوای یک طرفه استفاده گردید. یافته‌ها: نسبت VMO به VLL در هر دو گروه ورزشکاران مبتلا و غیر مبتلا به PFPS در تمامی زوایا تقریباً پایین‌تر از عدد 1 بوده و ورزشکاران سالم نسبت‌های پایین‌تر را دارا بودند. تفاوت معناداری در هیچکدام از زوایا بین گروه مبتلا و غیرمبتلا به‌دست نیامد. نتیجه‌گیری: به نظر می‌رسد فعالیت‌های ورزشی مانع تضعیف عضله VMO در مقایسه با VLL می‌گردد و نسبت VMO به VLL در ورزشکاران مبتلا و غیرمبتلا به سندرم درد پاتلوفمورال تأثیرپذیر از این سندرم نیست.

Title: مقایسه شاخص‌های تعادلی ورزشکاران حرفه‌ای مبتلا و غیرمبتلابه سندرم درد پاتلوفمورال

http://rehabilitationj.uswr.ac.ir/article-1-1044-fa.html

هدف: بررسیشاخص‌های تعادلی ورزشکاران حرفه‌ای مبتلا به درد پاتلوفمورال و مقایسه وضعیت تعادل آنها با ورزشکاران سالم هدف این پژوهش بوده است. روش بررسی: تحقیق غیر تجربی حاضر از نوع مقایسه‌ای مورد-شاهدی بود که 16 ورزشکار مرد تیم‌های ملی مبتلا به این سندرم با انتخاب ساده و هدفمند و 16 ورزشکار مرد سالم تیم‌های ملی که بر اساس متغیرهای وزن، قد، سن، رشته ورزشی و غالب بودن اندام تحتانی با گروه مورد جور شده بودند، در آن شرکت کردند. جهت ارزیابی تعادل از سیستم ثباتی بایودکس استفاده گردید و سه شاخص ثبات کلی، قدامی-خلفی و طرفی در دو وضعیت چشم باز و بسته روی پای غالب مورد ارزیابی قرار گرفت. از آزمون‌های آماری کولموگروف-اسمیرنوف و تی مستقل جهت تحلیل داده‌ها استفاده شد. یافته‌ها: در وضعیت چشم باز فقط شاخص تعادلی کلی (۰/۰۵=P) تفاوت معناداری را بین دو گروه نشان داد. همچنین در وضعیت چشم بسته هم شاخص تعادلی کلی (۰/۰۰۴=P) و هم شاخص قدامی-خلفی (۰/۰۱=P) تفاوت معناداری داشتند. اما تفاوت معناداری بین دیگر شاخص‌ها مشاهده نشد. نتیجه‌گیری: به نظر میرسد شاید شاخص های ثباتی ورزشکاران مبتلا به سندرم درد پاتلوفمورال، به علت کاهش فعالیت عضلانی ناشی از درد پاتلوفمورال، دستخوش اختلال و کاهش شده باشد.

Title: بررسی تأثیر آگاهی صریح قبلی بر یادگیری حرکتی افراد سالمند

http://rehabilitationj.uswr.ac.ir/article-1-996-fa.html

هدف: انجام مهارت‌های حرکتی و یادگیری آنها در سالمندان اهمیت ویژه‌ای دارد. مطالعه حاضر با هدف بررسی نوع یادگیری مطلوب در افراد سالمند و مقایسه اثربخشی دو استراتژی آگاهی صریح و تلویحی در سالمندان انجام شد. روش بررسی: در یک مطالعه تحلیلی و مقایسه‌ای جهت بررسی تأثیر آگاهی صریح بر یادگیری، یک وظیفه حرکتی سریالی و یک نرم‌افزار کامپیوتری مورد استفاده قرار گرفت. در طی این وظیفه حرکتی ۴ مربع با رنگ‌های مختلف بر صفحه نمایش‌گر ظاهر می‌‌شد که داوطلب به محض مشاهده هر مربع باید کلید تعریف شده مرتبط با آن را فشار می‌‌داد که در صورت واکنش صحیح، مربع بعدی ظاهر می‌شد. ۳۰ نفر سالمند سالم به صورت تصادفی به دو گروه تقسیم و طی دو روز به فاصله ۴۸ ساعت 10 بلوک حرکتی مشتمل بر ۸۰۰ تکرار مربع‌های رنگی را تمرین ‌کردند. در گروه اول یا تلویحی در ابتدای آزمون هیچ‌گونه توضیحی در مورد ترتیب ظهور محرک‌ها داده نمی‌شد، اما در گروه دوم یا یادگیری صریح در ابتدای آزمون به نمونه ها توضیحات کاملی داده می‌شد. داده‌ها با آزمون آماری تی مستقل و آنالیز واریانس مورد تحلیل قرار گرفت. یافته‌ها: در مجموع به طور معناداری، زمان انجام بلوک‌های حرکتی 10-7 در گروه سالمند صریح بیشتر ازسالمند تلویحی بود. اختلاف زمان بلوک دوم با بلوک‌های هشتم و دهم در هر دو گروه معنا‌دار بود که به ترتیب به‌معنی بهبود عملکرد و یادگیری بود، ولی بین دو گروه اختلاف معنا‌داری وجود نداشت. نتیجه‌گیری: آگاهی قبلی در کسب مهارت سالمندان نه تنها تأثیری بر میزان یادگیری و کسب مهارت ندارد، بلکه باعث کاهش قابلیت یادگیری افراد سالمند می‌گردد که این مسئله در طراحی و پروتکل توانبخشی افراد سالمند اهمیت خاصی پیدا می‌کند.

Title: اندازه‌گیری زاویه کوادریسپس توسط روش جدید و مقایسه آن با روش متداول گونیامتری و روش مرجع رادیوگرافی در مردان سالم

http://rehabilitationj.uswr.ac.ir/article-1-998-fa.html

هدف: هدف این مطالعه کاستن از خطای اندازه گیری زاویه کوادریسپس به روش رایج گونیامتری با استفاده از دستگاه و روش جدیدی است که در دانشگاه علوم بهزیستی و توانبخشی ارتقاء یافته است. روش بررسی: 40 مرد با 80 زانوی سالم در این مطالعه متدولوژیک مورد بررسی قرار گرفتند. اجرای طرح و جمع آوری داده ها توسط یک آزمونگر که طراح و سازنده دستگاه و روش جدید نیز می باشد انجام شد. زاویه کوادریسپس برای هر دو پای مردان شرکت کننده با سه روش رادیوگرافی، گونیامتری و روش جدید، اندازه‌گیری شد. روایی و پایایی دستگاه و روش جدید در مقابل روش رایج گونیامتری با در نظر گرفتن اندازه زاویه در روش مرجع رادیوگرافی مورد سنجش قرار گرفت. محاسبه ICC و آزمون‌های کولموگروف-اسمیرنوف، تی زوج و کرولی ماکلی جهت آنالیز داده‌ها بکار رفت. یافته‌ها: تکرار پذیری مطلق و نسبی برای اندازه گیری زاویه کوادریسپس بین روش جدید و رادیوگرافی ۰/۹۳۵ شد، در صورتیکه این مقایسه برای روش گونیامتری عدد ۰/۶۹۶ محاسبه شد. ICC بین سه بار اندازه گیری با روش جدید ۰/۹۷۴ محاسبه شد. نتیجه‌گیری: ارتباط بسیار قوی بین نتایج حاصل از روش جدید نسبت به رادیوگرافی بدست آمد، در صورتی که نتایج گونیامتری ارتباط ضعیف‌تری با رادیوگرافی داشت. تکرار پذیری در روش جدید با حفظ شرایط مطالعه، بسیار خوب بوده و لذا می‌توان این روش را به عنوان روشی نو در اندازه‌گیری اینگونه زوایا به کار برد.

Title: تاثیر موبیلیزاسیون عصب بر یافته‌های بالینی در افراد مبتلا به سندروم تونل کارپ

http://rehabilitationj.uswr.ac.ir/article-1-834-fa.html

هدف: در سال‌های اخیر استفاده از تکنیک‌های موبیلیزاسیون عصب در فیزیوتراپی گیرافتادگی‌‌های اعصاب محیطی مطرح شده است. در این مطالعه سعی شد تا تأثیر افزودن تکنیک‌های موبیلیزاسیون عصب به مدالیته‌های فیزیوتراپی بر جنبه‌های مختلف ارزیابی بالینی مبتلایان به سندرم تونل کارپ بررسی شود. روش بررسی: در این کار آزمائی بالینی، ۲۰ بیمار مبتلا به سندرم تونل کارپ به صورت تصادفی به دو گروه کنترل و درمان تقسیم شدند. در هر دو گروه به مدت ۴ هفته از اسپلینت استراحت، دستگاه تحریک الکتریکی پوستی و اولتراسوند استفاده شد. در گروه درمان، علاوه بر مدالیته‌های فیزیوتراپی از تکنیک‌های موبیلیزاسیون عصب استفاده شد. مقیاس شدت سمپتوم‌ها، مقیاس بصری درد، مقیاس وضعیت عملکردی، علامت فالن، تانسیون عصب مدین، و زمان‌های تأخیری هدایت دیستال حسی و حرکتی عصب مدین مورد ارزیابی قرار گرفت. آزمون‌های کولموگروف-اسمیرنوف، تی زوجی، تی مستقل و مک‌نمار برای تحلیل داده‌ها استفاده شد. یافته‌ها: مقیاس شدت سمپتوم‌ها، مقیاس بصری درد، تانسیون عصب مدین و علامت فالن در هر دو گروه کنترل (به ترتیب P=0/001, P=0/031, P=0/011, P=0/028) و درمان (به ترتیب ۰/۰۰۱>P>۰/۰۰۱, P>۰/۰۰۱, P>۰/۰۰۸, P) بهبود یافتند؛ اما مقیاس وضعیت عملکردی (۰٫۰۰۱>P) و زمان تأخیری دیستال حرکتی (۰/۰۱۵=P) تنها در گروه درمان بهبود یافت. تانسیون عصب مدین (۰/۰۰۱=P) و مقیاس وضعیت عملکردی (۰/۰۰۴=P) در گروه درمان بهبودی بیشتری را نشان داد؛ اما اختلاف معناداری بین دو گروه از لحاظ سایر متغیرها مشاهده نشد. نتیجه‌گیری: مدالیته‌های فیزیوتراپی و موبیلیزاسیون عصب هر یک تأثیر مفیدی بر یافته‌های ارزیابی ذهنی و فیزیکی سندرم تونل کارپ دارند. افزودن تکنیک‌های موبیلیزاسیون عصب به برنامه فیزیوتراپی معمول باعث افزایش اثرات سودمند درمانی می‌شود.

Title: ترس از حرکت/درد در دردهای عضلانی اسکلتی

http://rehabilitationj.uswr.ac.ir/article-1-807-fa.html

هدف: هدف از این مطالعه، بررسی مروری تأثیرات روانی درد مثل ترس از درد و ترس از حرکت روی عملکرد بیماران مبتلا به دردهای عضلانی-اسکلتی و نتایج توانبخشی آنان است. روش بررسی: مطالعه مروری یافته ها: ترس احساسی همگانی و نیرومند است که می تواند تأثیر زیادی روی رفتار انسان داشته باشد.رفتارهای ناشی از ترس روی نتایج توانبخشی بیماران مبتلا به درد عضلانی-اسکلتی اثرات منفی دارند. تعدادی از مطالعات مقطعی ثابت کرده اند که ارتباط مثبتی بین ترس زیاد از درد و افزایش شدت درد و ناتوانی وجود دارد. همچنین مطالعات طولی متعددی نشان داده اند که ترس زیاد از درد مقدمه بروز نتایج بالینی ضعیف است. وجود عقاید و افکار مخاطره آمیز در بیمار، روی ترس از حرکت/آسیب مجدد تأثیر می گذارد. این ترس منجر به بروز رفتارهای احترازی، عدم کاربرد، افسردگی و ناتوانی در بیمار می شود. ثابت شده که ترس از حرکت روی نتیجه توانبخشی کمر درد حاد و مزمن، سندرم خستگی مزمن و سندرم فیبرومیالژیا تأثیر منفی دارد. یافته‌ها: در مجموع مطالعات اخیر نشان می دهند که فیزیوتراپیست ها هنگام توانبخشی افراد مبتلا به دردهای عضلانی-اسکلتی، نباید از نقش مهمی که ترس از درد و رفتارهای احترازی در عملکرد بیماران دارند، غافل شده و باید این عوامل رفتاری و شناختی را مورد ارزیابی قرار دهند. در حال حاضر شواهد زیادی مبنی بر ارزیابی ترس از درد در بیماران مبتلا به دردهای عضلانی-اسکلتی وجود دارد.ارزیابی و بررسی ترس از درد توسط پرسش نامه هایی صورت می گیرد که اعتبار و تکرارپذیری آنها در مطالعات مختلف اثبات شده است. در بررسی های اخیر نشان داده شده که علاوه بر پاسخهای احترازی ترس، پاسخهای تحملی که از طریق فعالیت بیش از حد فیزیکی منجر به درد مزمن می شوند نیز در تداوم درد نقش دارند.همانطور که در بیماران مبتلا به دردهای عضلانی-اسکلتی ترس از حرکت وجود دارد، در بیماران مبتلا به بیماری های استرس مزمن وجود ترس از تلاش فکری اثبات شده است. نتیجه‌گیری: ترس از درد در برخی از بیماران می تواند به اندازه خود درد ناتوان کننده باشد. در نتیجه تلاش های دسته جمعی برای اصلاح تکنیک های ارزیابی و بررسی فعلی و گسترش مداخلات درمانی که بتوانند ترس از درد را بطور مؤثری کاهش دهند، لازم است.
